# Methemoglobinemia

**DOI:** 10.21980/J8PH1B

**Published:** 2022-10-15

**Authors:** Ibrahim Alagha, Ghadeer Doman, Shaza Aouthmanyzx

**Affiliations:** * Ohio University Heritage College of Osteopathic Medicine, Athens, OH; ^ King Abdulaziz University, College of Medicine, Department of Emergency Medicine, Jeddah, Saudi Arabia; † University of Toledo College of Medicine, Department of Emergency Medicine, Toledo, OH

## Abstract

**Audience:**

The targeted audience for this simulation are emergency medicine providers, including residents as well as advanced practice providers, to properly educate on recognizing, diagnosing, and managing methemoglobinemia.

**Introduction:**

Methemoglobinemia is a blood disorder characterized by the presence of ferric form of hemoglobin in the blood. This form of hemoglobin can carry oxygen but is unable to release it effectively causing a range of symptoms including headache, dizziness, nausea, and cyanosis. It is rarely congenital and mostly caused by the exposure to oxidizing agents, such as local anesthetics and quinolones.^1^ Normally, oxygen can bind to hemoglobin while it is in the ferrous state (Fe2+). In cases of methemoglobinemia, the heme iron configuration is converted from ferrous (Fe2+) to ferric (Fe3+), making it unable to bind to oxygen. As a result, normal ferrous hemes experience an increased affinity for oxygen causing a leftward shift in the oxygen dissociation curve. This in turn causes functional anemia due to reduced oxygen carrying capacity.^1^ Methemoglobinemia can result from exposure to different medications as well as environmental factors and presents like other disease processes including chronic obstructive pulmonary disease exacerbations. Congenital methemoglobinemia due to cytochrome b5 reductase deficiency is very rare, but the actual incidence is not known. Increased frequency of disease has been found in Siberian Yakuts, Athabaskans, Eskimos, and Navajo.^2^ Although it is also an unusual occurrence, acquired methemoglobinemia is much more frequently encountered than the congenital form.^1^

In a 10-year retrospective study looking at the incidence rate of topical anesthetic-induced methemoglobinemia, it was found that the overall prevalence was 0.035%. A major risk factor was hospitalization at the time of a procedure being performed. An increased risk was also seen with benzocaine-based anesthetics.^3^

**Educational Objectives:**

At the end of this simulation case, participants should be able to: 1) recognize shortness of breath, cyanosis and respiratory distress, and the difference between all of them based on the clinical presentation 2) identify the underlying cause of the condition by conducting a thorough history and physical 3) know how to identify and treat methemoglobinemia by ordering necessary labs and interventions and understand the pathophysiology leading to methemoglobinemia 4) recognize patient’s response to treatment and continue to reassess.

**Educational Methods:**

This is a high-fidelity simulation case that allows participants to evaluate and treat methemoglobinemia in a safe environment. The case is followed by a debriefing and small group discussion to review patient care skills, medical knowledge, interpersonal communication, practice-based learning, and improvement.

**Research Methods:**

The educational content and efficacy were evaluated by oral feedback and a debriefing session immediately after completion of the simulation. A 5-point Likert scale was sent out to participants pre-simulation and post-simulation. Questions on the survey included whether they felt confident in their ability to recognize methemoglobinemia, understood the physiology and causes of methemoglobinemia, and felt confident in their ability to treat methemoglobinemia.

**Results:**

Sixteen learners responded to the survey, consisting of EM residents and medical students. Post simulation, approximately 92% of EM residents answered agree or strongly agree in their ability to recognize and treat methemoglobinemia compared to pre-sim survey of about 62.5%. Post-simulation feedback also resulted in positive reception, and learners found it useful to run through an uncommonly seen case in the hospital. Results showed overall improvement in recognition and treatment of methemoglobinemia among residents and medical students.

**Discussion:**

This simulation improved recognition of methemoglobinemia including signs and symptoms associated with it. Proper management and treatment options were included such as administration of methylene blue. Overall, this simulation was helpful in teaching EM residents how to recognize, manage, and treat methemoglobinemia. In addition, post-simulation debriefing allowed further discussion among residents, which they found valuable.

**Topics:**

Methemoglobinemia, shortness of breath, cyanosis, respiratory distress, anemia, methemoglobin, oxygen dissociation curve, emergency medicine simulation.

## USER GUIDE

**Table:** 

**List of Resources:**	
Abstract	1
User Guide	2
Instructor Materials	5
Operator Materials	14
Debriefing and Evaluation Pearls	18
Simulation Assessment	20

**Learner Audience:**

Interns, Junior Residents, Senior Residents, Advanced Practice Providers (Pas, NPs)

**Time Required for Implementation:**

**Instructor Preparation:** 20–30 minutes

**Time for case:** 15–20 minutes

**Time for debriefing:** 10–20 minutes

**Recommended Number of Learners per Instructor:**

2–5

**Topics:**

Methemoglobinemia, shortness of breath, cyanosis, respiratory distress, anemia, methemoglobin, oxygen dissociation curve, emergency medicine simulation.

**Objectives:**

At the conclusion of the simulation case and after debriefing session, learner will be able to:

1. Recognize shortness of breath, cyanosis, and respiratory distress, and the difference between all of them based on the clinical presentation.
2. Identify the underlying cause of the condition by conducting a thorough history and physical.
3. Know how to identify and treat methemoglobinemia by ordering necessary labs and interventions and understand the pathophysiology leading to methemoglobinemia.
4. Recognize patient’s response to treatment and continue to reassess.

### Linked objectives and methods

It is important for emergency medicine physicians to use this high-fidelity simulation method to quickly diagnose methemoglobinemia and provide an appropriate treatment plan. Methemoglobinemia requires a high index of suspicion and presents similar to other common complaints we see in emergency medicine. Learners will need to recognize that the patient is in respiratory distress and cyanotic (Objective 1). They will need to obtain a detailed medical/surgical history and perform in-depth physical exam to recognize acrocyanosis, which will help them find the underlying cause (Objective 2). The patient’s past medical history of deep vein thromboses (DVTs) will distract the learner and may delay appropriate treatment. Appropriate diagnostic labs will need to be ordered, specifically Co-oximetry, which will lead the learner to the correct diagnosis of methemoglobinemia (Objective 3). If learners can recognize that the patient is presenting with methemoglobinemia, they will need to be able to treat it appropriately. Methemoglobinemia patients do not respond to supplementary oxygen and may deteriorate if the learner is unable to recognize it early on. Learner will need to order the appropriate intervention, such as methylene blue, for the patient to improve and will continue to reassess (Objective 4).

### Recommended pre-reading for instructor

- Cyanosis, Chapter 29. Marx J, Hockberger R, Walls R, eds*. Rosen’s Emergency Medicine: Concepts and Clinical Practice*. 7^th^ ed. Philadelphia: Mosby/Elsevier; 2010:211–216.
- Jeffery WH, Zelicoff AP, Hardy WR. Acquired methemoglobinemia and hemolytic anemia after usual doses of phenazopyridine. *Drug Intell Clin Pharm*. 1982 Feb;16(2):157–9.
- Any emergency medicine text on the presentation, diagnosis, and treatment of methemoglobinemia.

### Results and tips for successful implementation

This case was made for a high-fidelity simulation scenario to allow learners to recognize and treat methemoglobinemia. This simulation was used on emergency medicine interns and residents. Learners were assessed during the simulation and given graded percentages, which were later discussed during the debriefing session. Attending EM physicians were used during this simulation to evaluate learners’ performance. There was also a senior resident in the room acting as a confederate to help move the case along. It is recommended to provide a pre-simulation survey as well as a post-simulation survey to get a baseline reading of where learners are at. During our simulation, 16 EM residents responded to both a pre-simulation as well as a post-simulation survey consisting of 6 questions on a 5-point Likert scale (questions included below). Post simulation, approximately 92% of EM residents (12 learners) answered agree or strongly agree in their ability to recognize and treat methemoglobinemia compared to pre-sim survey of about 62.5% (10 learners). Post-simulation feedback also resulted in positive reception and learners found it useful to run through an uncommonly seen case in the hospital. 10 medical students also responded to our survey. Results showed overall improvement in recognition and treatment of methemoglobinemia among residents and medical students.

1. I feel confident in my ability to recognize and differentiate between ventilation and oxygenation.
2. I feel confident in my ability to recognize a methemoglobinemia presenting patient.
3. I feel confident in my ability to recognize carbon monoxide poisoning.
4. I feel confident in my ability to treat and manage methemoglobinemia.
5. I feel confident in my ability to explain methemoglobinemia to a patient.
6. I feel confident in my ability to recognize the causes of methemoglobinemia.

Overall feedback from learners and instructors was positive. This was measured using pre- and post-simulation surveys asking about the learners’ ability to differentiate between ventilation and oxygenation. They were also asked about their ability to recognize and treat methemoglobinemia. Lastly, learners were surveyed about their ability to explain methemoglobinemia to a patient.

## Supplementary Information























## Figures and Tables

**Table:** 

#1	Electrocardiogram (ECG)
#2	Chest Radiograph (CXR)
#3	Complete Blood Count (CBC)
#4	Comprehensive Metabolic Panel (CMP) with phosphorus and magnesium
#5	Troponin
#6	Arterial Blood Gas (ABG) with Methemoglobin
#7	Lactate

**Table:** 

**Vitals:**	Heart Rate (HR) 113	Blood Pressure (BP) 107/75 right arm
	Respiratory Rate (RR) 24	Temperature (T) 37.5°F
	Oxygen Saturation (O_2_Sat) 82% on room air	

**Figure:**
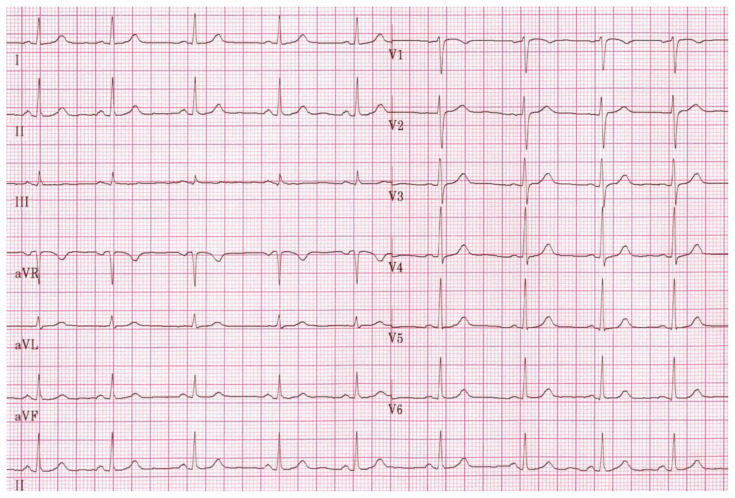


**Figure:**
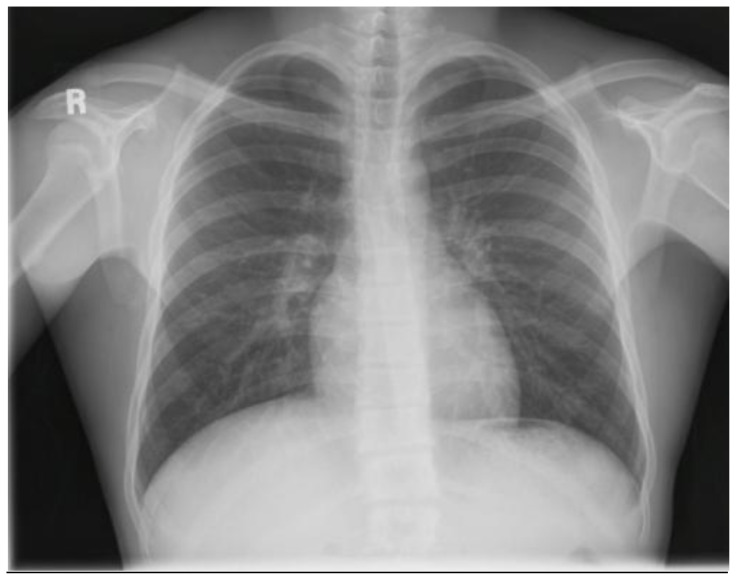


**Table:** 

*Complete blood count (CBC)*	
White blood count (WBC)	6.07 × 1000/mm^3^
Red Blood Count (RBC)	5.75 × 10^6^/mm^3^
Hemoglobin (Hgb)	16.6 g/dL
Hematocrit (HCT)	47.7%
Mean Corpuscular Volume (MCV)	83.0 fL
Mean Corpuscular Hemoglobin (MCH)	28.9 pg
Mean Corpuscular Hemoglobin Concentration (MCHC)	34.8 g/dL
RDW	12.6%
Platelet (Plt)	239 × 1000/mm^3^

**Table:** 

*Comprehensive metabolic panel (CMP)*	
Sodium	141 mEq/L
Potassium	4.1 mEq/L
Chloride	106 mEq/L
CO2	24 mmol/L
Anion gap	11 mmol/L
Blood Urea Nitrogen (BUN)	11 mg/dL
Creatinine (Cr)	0.63 mg/dL
Glucose	121 mg/dL
Calcium	9.0 mg/dL
Total Protein	6.1 g/dL
Albumin	51 U/L
Total bilirubin	0.6 mg/dL
Aspartate aminotransferase (AST)	28 units/L
Alanine aminotransferase (ALT)	10 units/L
Phosphorous	3.5 mg/dL
Magnesium	2.0 mEq/L

**Table:** 

*Troponin*	<0.01 ng/mL

**Table:** 

*Arterial Blood Gas (ABG)*	
pH	7.40
pCO2	47 mEq/L
paO2	104 mmHg
Bicarbonate (HCO_3_)	20 mEq/L
MetHb	42%

**Table:** 

*Lactate*	3.4 mmol/L

**Table:** SIMULATION EVENTS TABLE:

Minute (state)	Participant action/ trigger	Patient status (simulator response) & operator prompts	Monitor display (vital signs)
**State 1: Arrival to ED**	Patient arrived to the ED in respiratory distress. Participant should request patient to be placed on supplementary oxygen after identifying respiratory distress. Learner should identify respiratory distress. Learner should obtain past medical and surgical hx to think about the possibility of having Methemoglobinemia 2/2 local anesthetics. Learner should complete physical exam. When learner completes all these steps, you may proceed to state 2.	Patient is tachycardic and in respiratory distress. Airway intact. Physical exam shows mild wheezing bilaterally along with cyanotic fingertips. He does appear anxious. When the patient is placed on non-rebreather mask and given breathing treatment, proceed to next step. If patient is not placed on a non-rebreather mask within 3 minutes, RR will increase to 30 with HR increasing to 125 bpm. Patient should be speaking 2–3-word sentences but is otherwise awake and alert. Labs can be ordered in this step. Do not continue to next step if supplementary oxygen is not ordered. If the learner completely fails to order labs and/or place patient on supplementary oxygen, nurse in the room or RT may bring it to their attention that the patient is exhibiting acrocyanosis and is hypoxic.	T: 37.5°C HR: 113 BP: 107/75 RR: 24 O2: 85%
**State 2: (03:00)**	Participant should identify continued respiratory distress without any improvement. RR has increased as well as HR. Learner should attempt to use BiPAP since nonrebreather did not help. (+SIRS criteria as well as PMHx of DVT will most likely distract the learner.) Participant should create a differential diagnosis. Only labs ordered should result. Continue to State 3 when methemoglobin level is ordered. If it is not ordered, proceed to State 4.	Patient remains tachypneic without any response to supplementary oxygen or BiPAP if ordered. Wheezing is bilateral and worsening. If learner does not recognize methemoglobinemia yet, the nurse may prompt the learner that the patient’s blood appears “chocolate colored” and patient is becoming confused.	T: 37.5°C HR: 125 BP: 106/78 RR: 30 O2: 82%
**State 3: (06:00)**	Learner should recognize methemoglobinemia if it has resulted. Methylene blue should be ordered. May proceed to State 5.	Patient should be given Methylene blue. When methylene blue is given, patient’s breathing should improve. Normal coloration of the distal extremities should be observed as well.	T: 37.5°C HR: 97 BP: 128/80 RR: 18 O2: 95%
**State 4: (06:00)**	Only continue in this state if methemoglobinemia is not recognized or if patient is intubated. Patient will deteriorate and go into respiratory arrest. If methemoglobinemia is recognized, proceed to State 5.	Patient will begin to lose consciousness and have a seizure. He will then go into respiratory arrest. Resuscitation efforts may begin. Patient will not have ROSC unless methylene blue is given. If methemoglobinemia is recognized at this time, you may go back to State 3.	T: 37°C HR: 50 BP: 72/50 RR: None O2: 56%
**State 5: (10:00)**	Learner should continue monitoring the patient. Patient should be admitted at this time.	Patient will show some improvement after the medication. Repeat MetHb level to see if more doses are needed. CASE CLOSED	T: 37.5°C HR: 88 BP: 128/80 RR: 16 O2: 97%

**Table:** 

**Critical Actions:** Place patient on the monitor Obtain full history from the patient Order proper labs Place patient on supplementary oxygen Order Co-Oximetry when patient does not respond Establish diagnosis of Methemoglobinemia Administer methylene blue Admit patient	0:00

**Table:** 

	Milestone	Did not achieve level 1	Level 1	Level 2	Level 3
1	**Emergency Stabilization (PC1)**	□ Did not achieve Level 1	□ Recognizes abnormal vital signs	□ Recognizes an unstable patient, requiring intervention Performs primary assessment Discerns data to formulate a diagnostic impression/plan	□ Manages and prioritizes critical actions in a critically ill patient Reassesses after implementing a stabilizing intervention
2	**Performance of focused history and physical (PC2)**	□ Did not achieve Level 1	□ Performs a reliable, comprehensive history and physical exam	□ Performs and communicates a focused history and physical exam based on chief complaint and urgent issues	□ Prioritizes essential components of history and physical exam given dynamic circumstances
3	**Diagnostic studies (PC3)**	□ Did not achieve Level 1	□ Determines the necessity of diagnostic studies	□ Orders appropriate diagnostic studies. Performs appropriate bedside diagnostic studies/procedures	□ Prioritizes essential testing Interprets results of diagnostic studies Reviews risks, benefits, contraindications, and alternatives to a diagnostic study or procedure
4	**Diagnosis (PC4)**	□ Did not achieve Level 1	□ Considers a list of potential diagnoses	□ Considers an appropriate list of potential diagnosis May or may not make correct diagnosis	□ Makes the appropriate diagnosis Considers other potential diagnoses, avoiding premature closure
5	**Pharmacotherapy (PC5)**	□ Did not achieve Level 1	□ Asks patient for drug allergies	□ Selects an medication for therapeutic intervention, consider potential adverse effects	□ Selects the most appropriate medication and understands mechanism of action, effect, and potential side effects Considers and recognizes drug-drug interactions
6	**Observation and reassessment (PC6)**	□ Did not achieve Level 1	□ Reevaluates patient at least one time during case	□ Reevaluates patient after most therapeutic interventions	□ Consistently evaluates the effectiveness of therapies at appropriate intervals
7	**Disposition (PC7)**	□ Did not achieve Level 1	□ Appropriately selects whether to admit or discharge the patient	□ Appropriately selects whether to admit or discharge Involves the expertise of some of the appropriate specialists	□ Educates the patient appropriately about their disposition Assigns patient to an appropriate level of care (ICU/Tele/Floor) Involves expertise of all appropriate specialists
9	**General Approach to Procedures (PC9)**	□ Did not achieve Level 1	□ Identifies pertinent anatomy and physiology for a procedure Uses appropriate Universal Precautions	□ Obtains informed consent Knows indications, contraindications, anatomic landmarks, equipment, anesthetic and procedural technique, and potential complications for common ED procedures	□ Determines a back-up strategy if initial attempts are unsuccessful Correctly interprets results of diagnostic procedure
20	**Professional Values (PROF1)**	□ Did not achieve Level 1	□ Demonstrates caring, honest behavior	□ Exhibits compassion, respect, sensitivity and responsiveness	□ Develops alternative care plans when patients’ personal beliefs and decisions preclude standard care
22	**Patient centered communication (ICS1)**	□ Did not achieve level 1	□ Establishes rapport and demonstrates empathy to patient (and family) Listens effectively	□ Elicits patient’s reason for seeking health care	□ Manages patient expectations in a manner that minimizes potential for stress, conflict, and misunderstanding. Effectively communicates with vulnerable populations, (at risk patients and families)
23	**Team management (ICS2)**	□ Did not achieve level 1	□ Recognizes other members of the patient care team during case (nurse, techs)	□ Communicates pertinent information to other healthcare colleagues	□ Communicates a clear, succinct, and appropriate handoff with specialists and other colleagues Communicates effectively with ancillary staff

## INSTRUCTOR MATERIALS

**Case Title:** Methemoglobinemia

**Case Description & Diagnosis (short synopsis):** It is 9 pm, and you have a 55-year-old male patient brought by EMS from home complaining of worsening shortness of breath, chills, lightheadedness, headache, and palpitation that started a few hours prior to arrival. Patient had dental work done this morning (this will be mentioned if learner asked about past surgical history), where he had local 20% Benzocaine used during the treatment at the dental clinic. Patient will have a constant Spo2 of 82%, as well as mild wheezing, and you notice that he is coughing too. Patient will be respiratory distress and have acrocyanosis.

**Equipment or Props Needed:**

- High-Fidelity Simulator
- Adult Nasal Cannula
- Intubation Supplies: endotracheal tube, Stylette, Mac/Miller Blade, bougie, syringe, colorimetric capnography
- Adult non-rebreather mask
- Adult bag-valve mask
- Ventilator

**Confederates needed:**

Nurse and/or family may be in the room to provide history.

**Stimulus Inventory:**

**Background and brief information:** 55-year-old male patient brought into the emergency department via EMS from home in respiratory distress and complaining of shortness of breath, chills, lightheadedness, headache, and palpitations that have been going on for a few hours.

**Initial presentation:** Patient is in respiratory distress with mild wheezing on exam and coughing. Acrocyanosis is appreciated as well.

**How the scene unfolds:** Patient presents to the emergency department with respiratory distress. Participants should place patient on oxygen and obtain vitals while taking history. While taking history, participant should ask about any surgical history, and the patient will inform them that he had a dental procedure done earlier that morning and had 20% benzocaine used during treatment. Patient’s oxygen saturation should stay constant at 85% along with mild wheezing and a cough. These symptoms will distract the participant and drag him toward underlying COPD that the patient has. In addition, patient will have acrocyanosis, which should guide towards a correct diagnosis. Participant should perform a physical exam, specifically cardiovascular and pulmonary. Labs, ECG, and imaging might be ordered at this time along with a breathing treatment. Participant should attempt to use BiPAP, which will not help his condition. Patient’s tachycardia will continue to worsen as well as his tachypnea. His hypoxia will not improve and will only worsen if learner does not recognize methemoglobinemia. A nurse confederate may guide the learner and inform him that the patient’s blood appears “chocolate colored.” If participant suspects methemoglobinemia, co-oximetry should be ordered to correctly identify methemoglobinemia. After participant identifies methemoglobinemia, methylene blue should be ordered and a repeat MetHb should be ordered. Failure to correctly identify methemoglobinemia will cause the patient to become confused and suffer a seizure. Patient will eventually go into respiratory arrest if methemoglobinemia is not recognized.

**Critical actions:**

1. Place patient on monitor and obtain vital signs
  - Place on supplementary oxygen
2. Obtain full history from patient, including surgical history
3. Order labs, imaging, and ECG
4. Recognize patient is not improving with supplementary oxygen and suspect other causes
5. Order co-oximetry level to detect methemoglobinemia
6. Administer methylene blue for methemoglobinemia treatment
7. Admit patient

**Case Title:** Methemoglobinemia

**Chief Complaint:** Shortness of breath, chills, lightheadedness, headache, and palpations that has been going on for a few hours. Patient has a history of COPD.

**General Appearance:** Appears in distress

**Primary Survey:**

- **Airway:** Clear. Patient speaking 2–3-word sentences.
- **Breathing:** Bilateral breath sounds. Bilateral mild wheezing. Tachypneic.
- **Circulation:** Tachycardic.

**History:**

- **History of present illness:** A 55-year-old male patient brought into the emergency department via EMS from home in respiratory distress complaining of shortness of breath, chills, lightheadedness, headache, and palpitations that have been going on for a few hours.
- **Past medical history:** COPD, HTN, DM, Dyslipidemia, Prior DVT
- **Past surgical history:** Appendectomy and dental procedure this morning (only provided if asked)
- **Patient’s medications:** inhaler, steroids, metformin, lisinopril, and aspirin
- **Allergies:** None
- **Social history:** Current every day smoker. No alcohol or illicit drug use
- **Family history:** Irrelevant

**Secondary Survey/Physical Examination:**

- **General appearance:** In distress
- **HEENT:**
  ○ **Head:** Within normal limits
  ○ **Eyes:** Within normal limits
  ○ **Ears:** Within normal limits
  ○ **Nose:** Within normal limits
  ○ **Throat:** Within normal limits
- **Neck:** Within normal limits
- **Heart:** Tachycardic otherwise within normal limits.
- **Lungs:** Mild wheezing bilaterally. Tachypneic.
- **Abdominal/GI:** Within normal limits
- **Genitourinary:** Within normal limits
- **Rectal:** Within normal limits
- **Extremities:** Within normal limits
- **Back:** Within normal limits
- **Neuro:** Within normal limits
- **Skin:** Acrocyanosis (should specifically ask)
- **Lymph:** Within normal limits
- **Psych:** Within normal limits

*ECG*

de Jong J. *Normal Tracing*.; 2011. https://en.ecgpedia.org/wiki/Normal_Tracing. Accessed September 30, 2022. CC BY-NC-SA 3.0.

*Initial Chest X-Ray*

Stillwaterising. In Wikimedia Commons.

https://commons.wikimedia.org/wiki/File:Chest_Xray_PA_3-8-2010.png. Published March 8, 2010. Accessed September 29, 2021. CC BY 1.0.

## OPERATOR MATERIALS

**Diagnosis:**

Methemoglobinemia

**Disposition:**

Admit patient

## DEBRIEFING AND EVALUATION PEARLS

### Methemoglobinemia

#### Background/Causes

Normally, oxygen can bind to hemoglobin while it is in the ferrous state (Fe2+). In cases of methemoglobinemia, the heme iron configuration is converted from ferrous (Fe2+) to ferric (Fe3+), making it unable to bind to oxygen. As a result, normal ferrous hemes experience an increased affinity for oxygen causing a leftward shift in the oxygen dissociation curve. This in turn causes functional anemia due to reduced oxygen carrying capacity.^1^ Methemoglobinemia can result from exposure to different medications as well as genetic, dietary, and idiopathic sources.^4^ It presents similar to other disease processes including COPD exacerbations. Methemoglobinemia should be suspected in any patient with cyanosis and not responsive to supplemental O2.

Methemoglobinemia has two primary causes, acquired and congenital. Although it is also an unusual occurrence, acquired methemoglobinemia is much more frequently encountered than the congenital form.^1^ Most commonly, it is caused by ingestion or skin exposure to an oxidizing agent, such as benzocaine and/or other local anesthetics.^4^ It can also be caused by nitrites, nitrates, quinolones, antimalarials, or Dapsone.^5^ In a 10-year retrospective study looking at the incidence rate of topical anesthetic-induced methemoglobinemia, it was found that the overall prevalence was 0.035%. A major risk factor was hospitalization at the time of a procedure being performed. An increased risk was also seen with benzocaine-based anesthetics, causing up to two-thirds of cases.^3^,^6^ This has led to the recommendation that benzocaine should no longer be used and prilocaine should not be used in children younger than 6 months, in pregnant women, or in patients taking other oxidizing drugs.^6^

Very rarely, methemoglobinemia maybe congenital in nature due to NADH reductase resulting in the inability to reduce ferric to ferrous iron (Fe3+ to Fe2+).^7^ Congenital methemoglobinemia due to cytochrome b5 reductase deficiency is also very rare, but the actual incidence is not known. Increased frequency of congenital methemoglobinemia has been found in Siberian Yakuts, Athabaskans, Eskimos, and Navajo.^2^

#### Clinical Presentation

Patients with methemoglobinemia may represent with a variety of symptoms depending on the amount of methemoglobinemia in the blood. In less severe cases, patients may present with cyanosis. As levels progress, additional symptoms develop including anxiety, lightheadedness, headache, tachycardia, fatigue, confusion, dizziness, tachypnea, arrhythmias, seizures, and coma.^4^ Patients may also develop lactic acidosis and myocardial ischemia. For this reason, learners should obtain labs including CBC, CMP, venous blood gas or ABG if possible. ECG should also be obtained to look for signs of ischemia.

Methemoglobinemia is a clinical diagnosis based on history and presenting symptoms including hypoxemia refractory to supplementary oxygen.^1^ Diagnosing methemoglobinemia requires high index of suspicion. Failure for the patient to respond appropriately to BiPAP and other supplementary oxygen should allow learners to explore other options. Co-oximetry may be obtained, which will reveal elevated levels of methemoglobin. The existence of an underlying heart, lung, or blood disease may exacerbate the toxicity of methemoglobinemia.^4^ Obtaining a past medical history can be very vital in this case because it may guide them towards methemoglobinemia.

#### Treatment/Management

- First step of management is to reduce exposure to known toxin if actively present.
- High-flow oxygen, nonrebreather, and BiPAP may improve symptoms, but pulse oximeter reading will remain unchanged.
- Methylene blue should be given upon recognizing methemoglobinemia. Correct dosing is 1–2mg/kg IV over 5 minutes. This dose may be repeated if patient does not improve within 30 minutes.^4^
  ○ In patients with contraindication for methylene blue, such as G6PD deficiency, moderate amounts of vitamin C, 300–1000mg/day orally, may be used in divided doses. If ineffective, exchange transfusion may be completed.
- Dextrose can also be given. This is due to the catabolism of sugar being a major source of NADH in the RBC. For reducing enzymes to be effective, glucose must be available in adequate supply. Dextrose is also necessary to form NADPH, which is necessary for methylene blue to be effective. Specific data about the amounts of dextrose to be given are not available but the recommendation is to give maintenance amounts in normoglycemia patients and standard dextrose therapy should be given to hypoglycemic patients.^4^
- Hyperbaric Oxygenation Monotherapy has been successfully used before and may be used if patient fails methylene blue, or they are contraindicated.^8^
- IV fluids should also be given along with bicarbonate in cases of metabolic acidosis.^5^

#### Other debriefing points

- Discuss evaluation and treatment of methemoglobinemia
- Emphasize the importance of obtaining relevant past medical/surgical history

## SIMULATION ASSESSMENT

*Methemoglobinemia*

Learner: _________________________________________

***Assessment Timeline***

This timeline is to help observers assess their learners. It allows observer to make notes on when learners performed various tasks, which can help guide debriefing discussion.

**Critical Actions:**

□ Place patient on the monitor
□ Obtain full history from the patient
□ Order proper labs
□ Place patient on supplementary oxygen
□ Order Co-Oximetry when patient does not respond
□ Establish diagnosis of Methemoglobinemia
□ Administer methylene blue
□ Admit patient

**Summative and formative comments:**

**Milestones assessment:**
