# Supplementary material for: Methemoglobinemia
Source: J Educ Teach Emerg Med. 2022 Oct 15;7(4):S1–S26. doi: 10.21980/J8PH1B (PMC10332666; doi:10.21980/J8PH1B)
Supplement: Supplementary file 1 [file JETem-7-4-S1-supp1.pptx]

## Slide 1
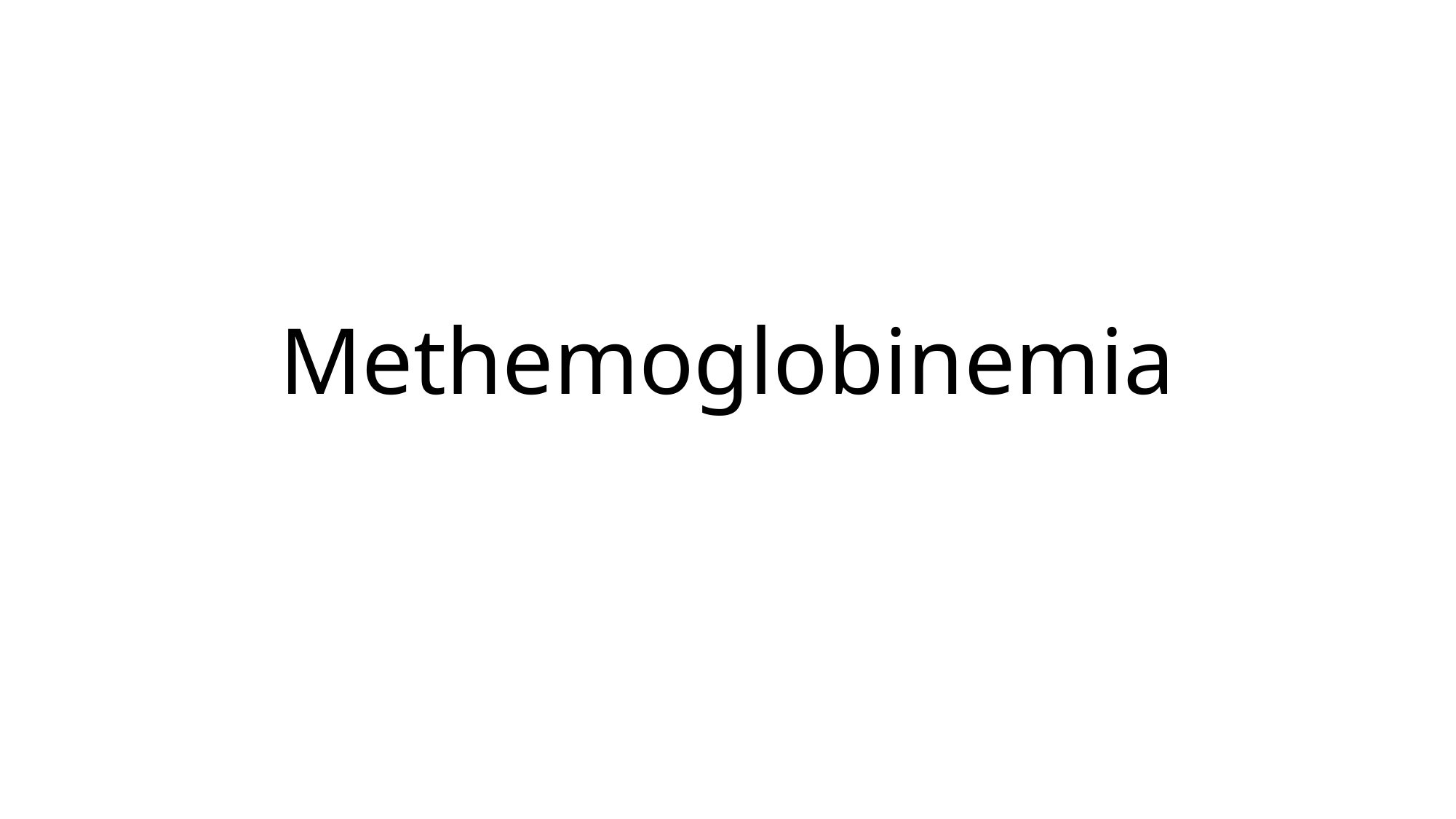

# Methemoglobinemia

## Slide 2
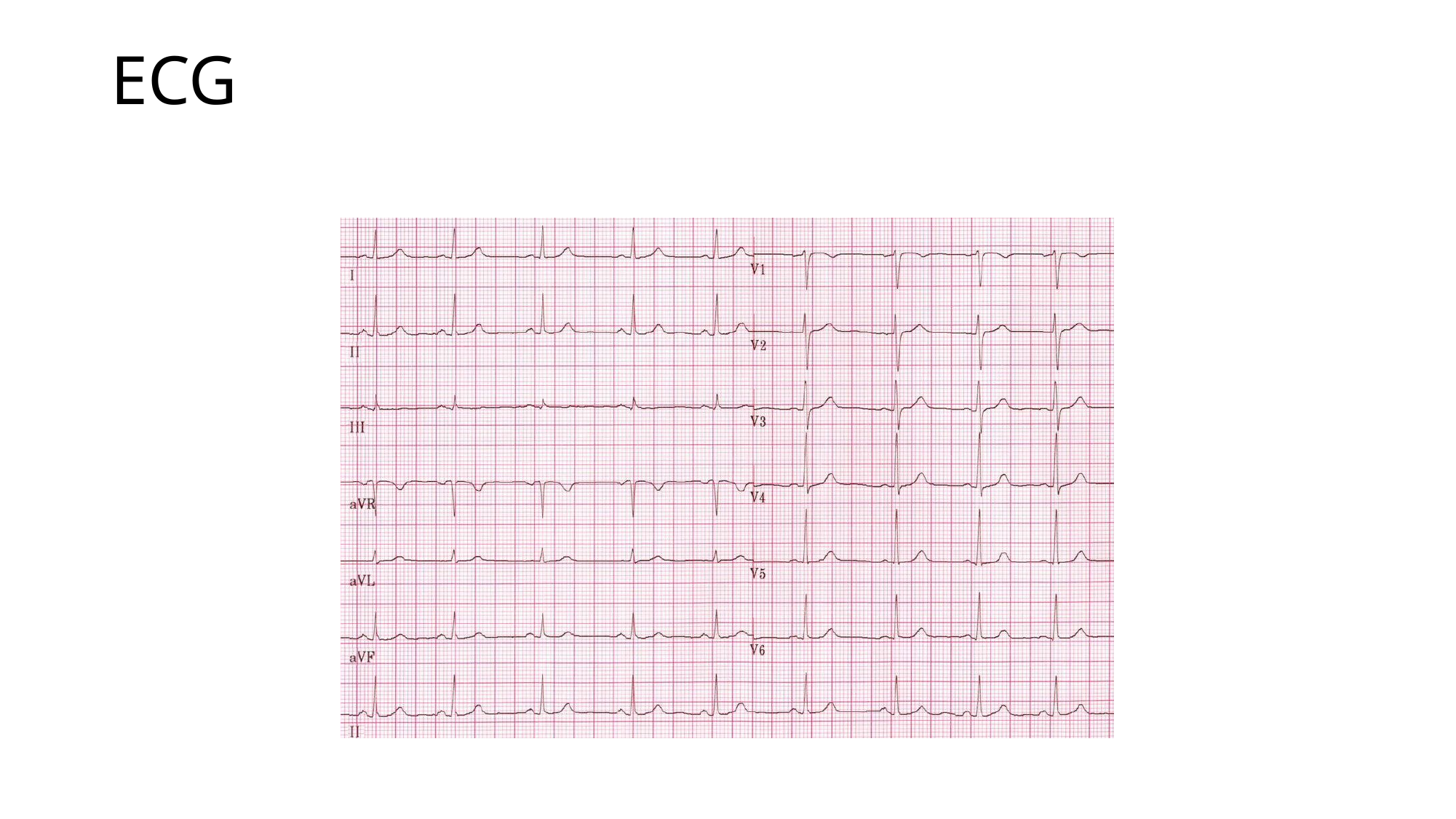

# ECG

## Slide 3
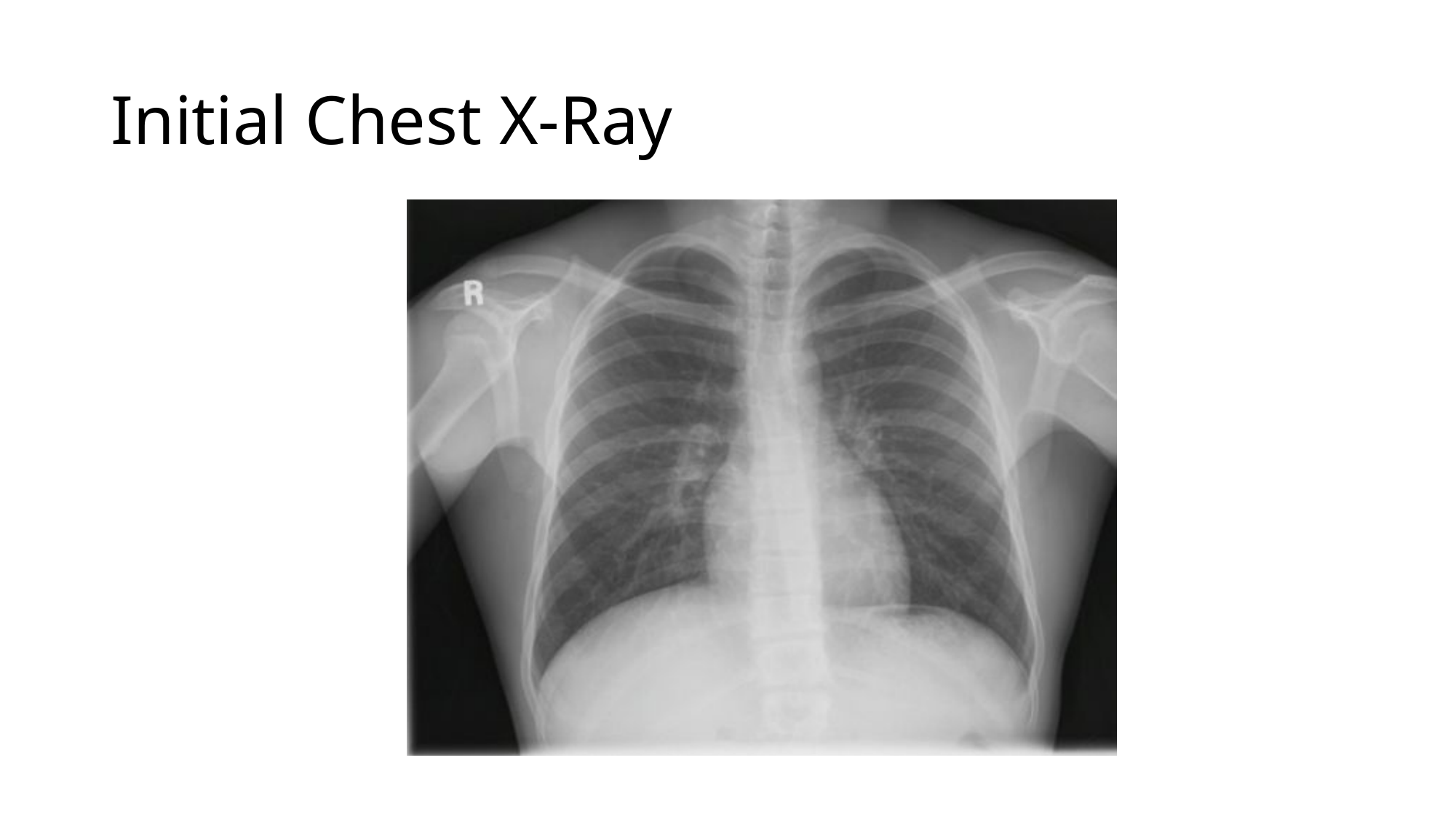

# Initial Chest X-Ray

## Slide 4
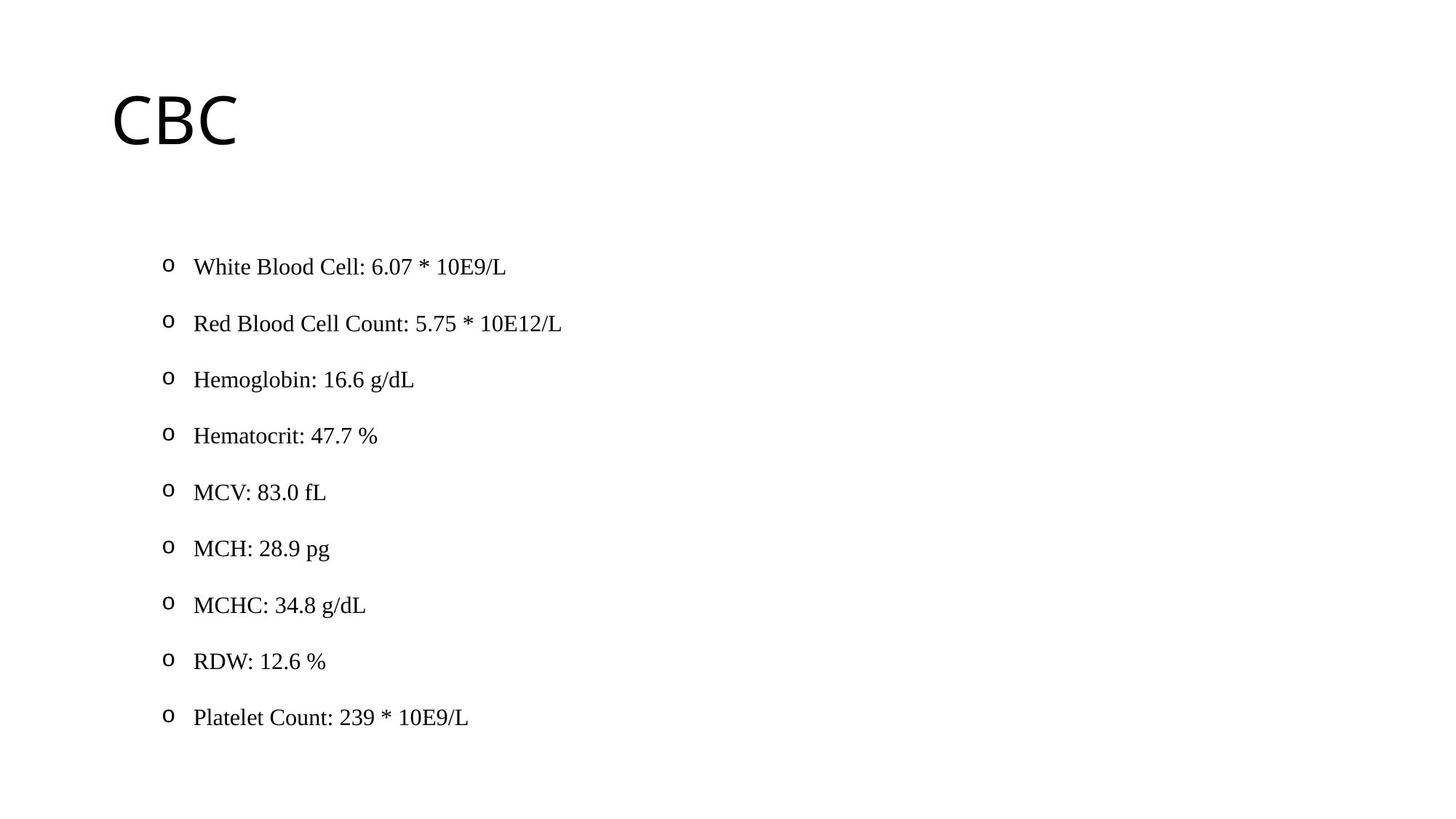

# CBC
White Blood Cell: 6.07 * 10E9/L
Red Blood Cell Count: 5.75 * 10E12/L
Hemoglobin: 16.6 g/dL
Hematocrit: 47.7 %
MCV: 83.0 fL
MCH: 28.9 pg
MCHC: 34.8 g/dL
RDW: 12.6 %
Platelet Count: 239 * 10E9/L

## Slide 5
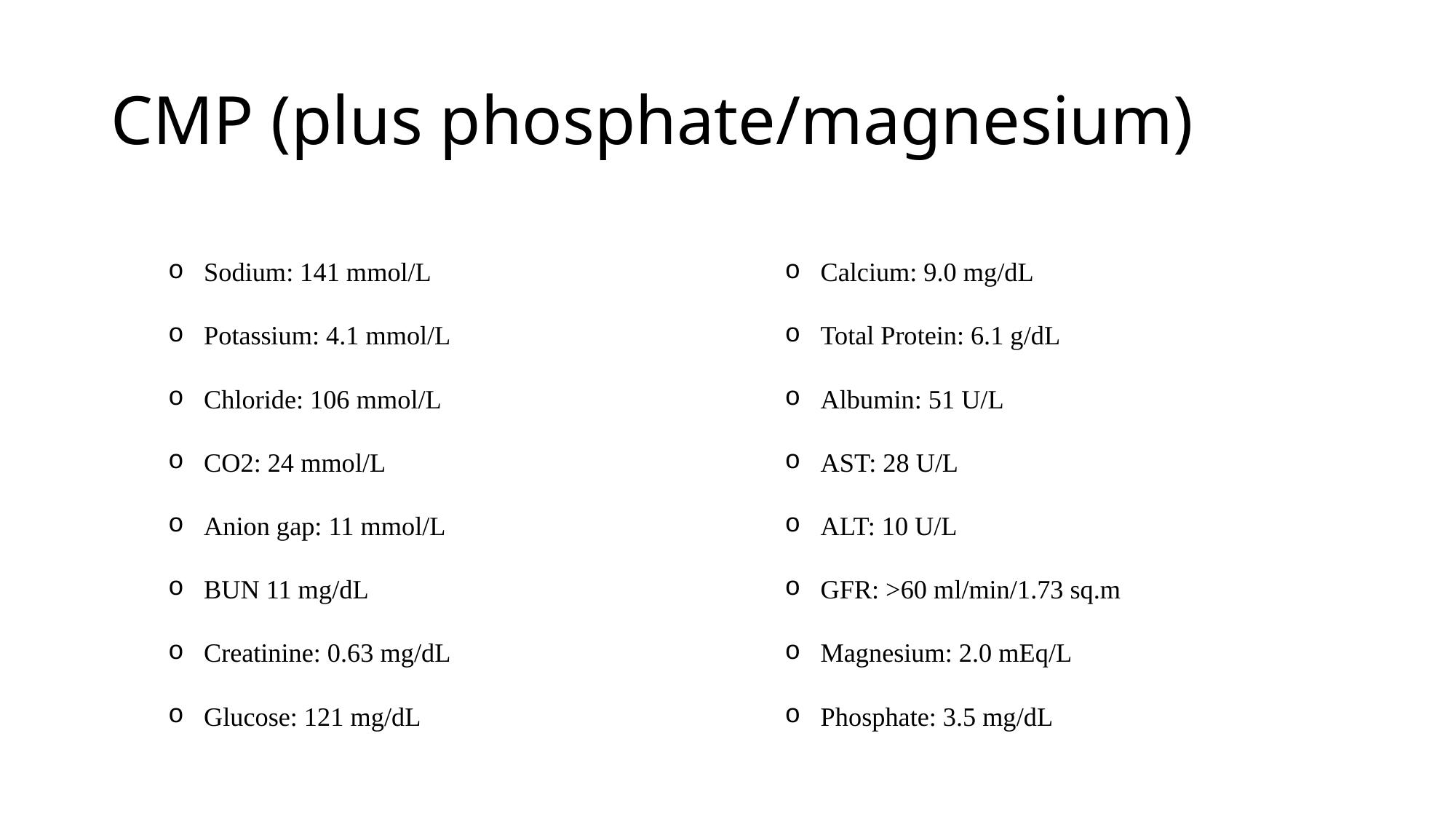

# CMP (plus phosphate/magnesium)
Sodium: 141 mmol/L
Potassium: 4.1 mmol/L
Chloride: 106 mmol/L
CO2: 24 mmol/L
Anion gap: 11 mmol/L
BUN 11 mg/dL
Creatinine: 0.63 mg/dL
Glucose: 121 mg/dL
Calcium: 9.0 mg/dL
Total Protein: 6.1 g/dL
Albumin: 51 U/L
AST: 28 U/L
ALT: 10 U/L
GFR: >60 ml/min/1.73 sq.m
Magnesium: 2.0 mEq/L
Phosphate: 3.5 mg/dL

## Slide 6
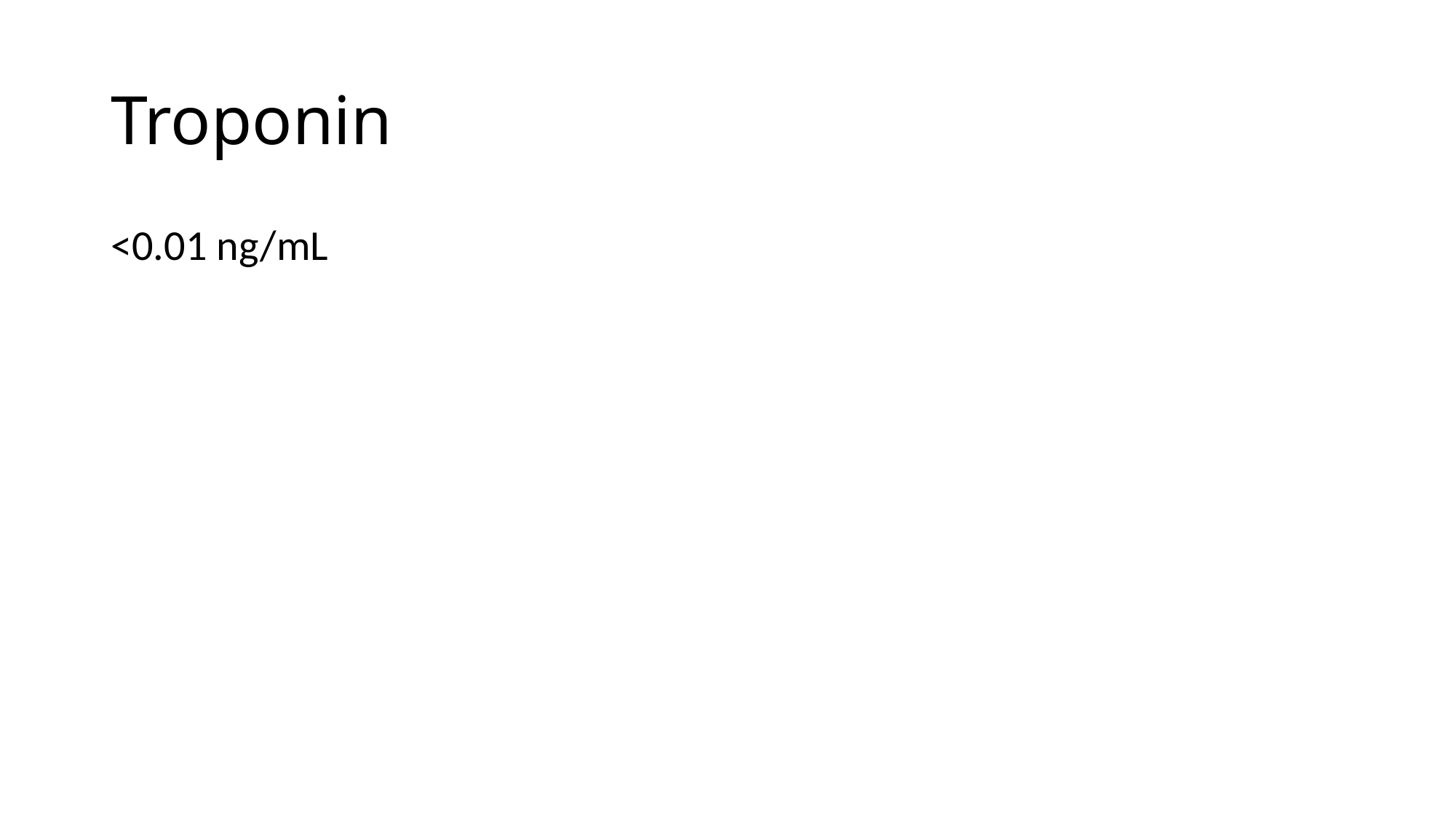

# Troponin
<0.01 ng/mL

## Slide 7
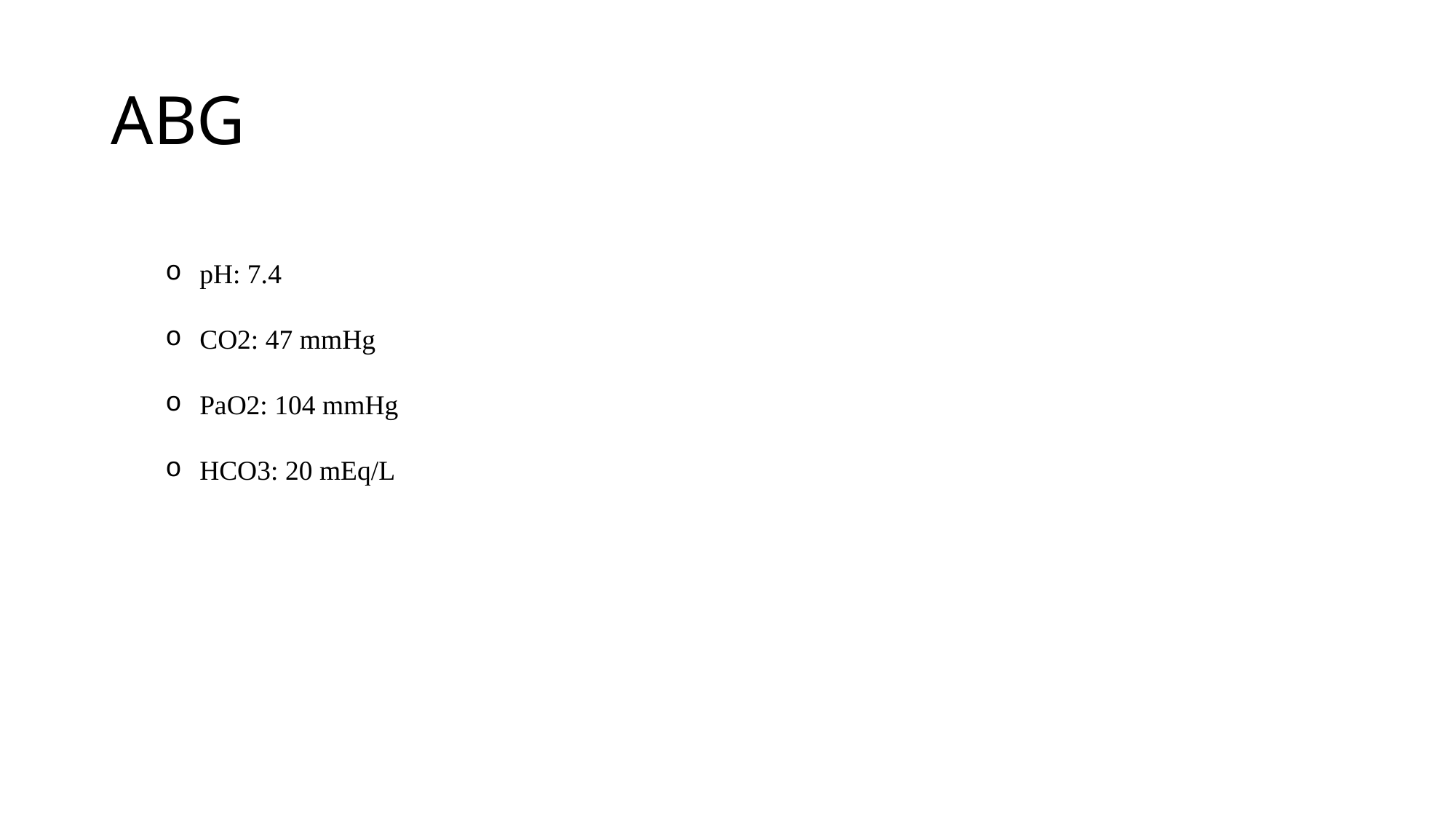

# ABG
pH: 7.4
CO2: 47 mmHg
PaO2: 104 mmHg
HCO3: 20 mEq/L

## Slide 8
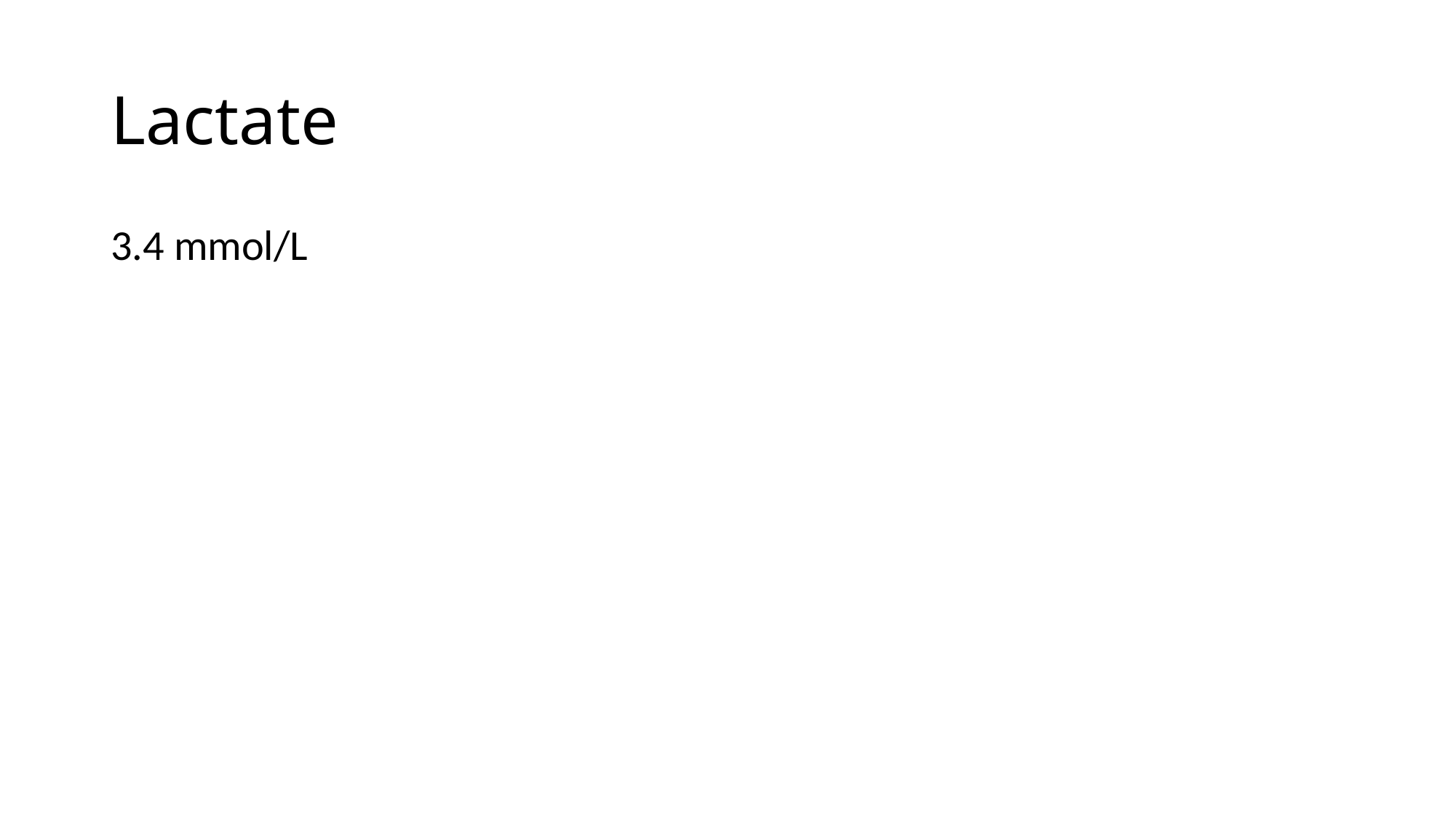

# Lactate
3.4 mmol/L

## Slide 9
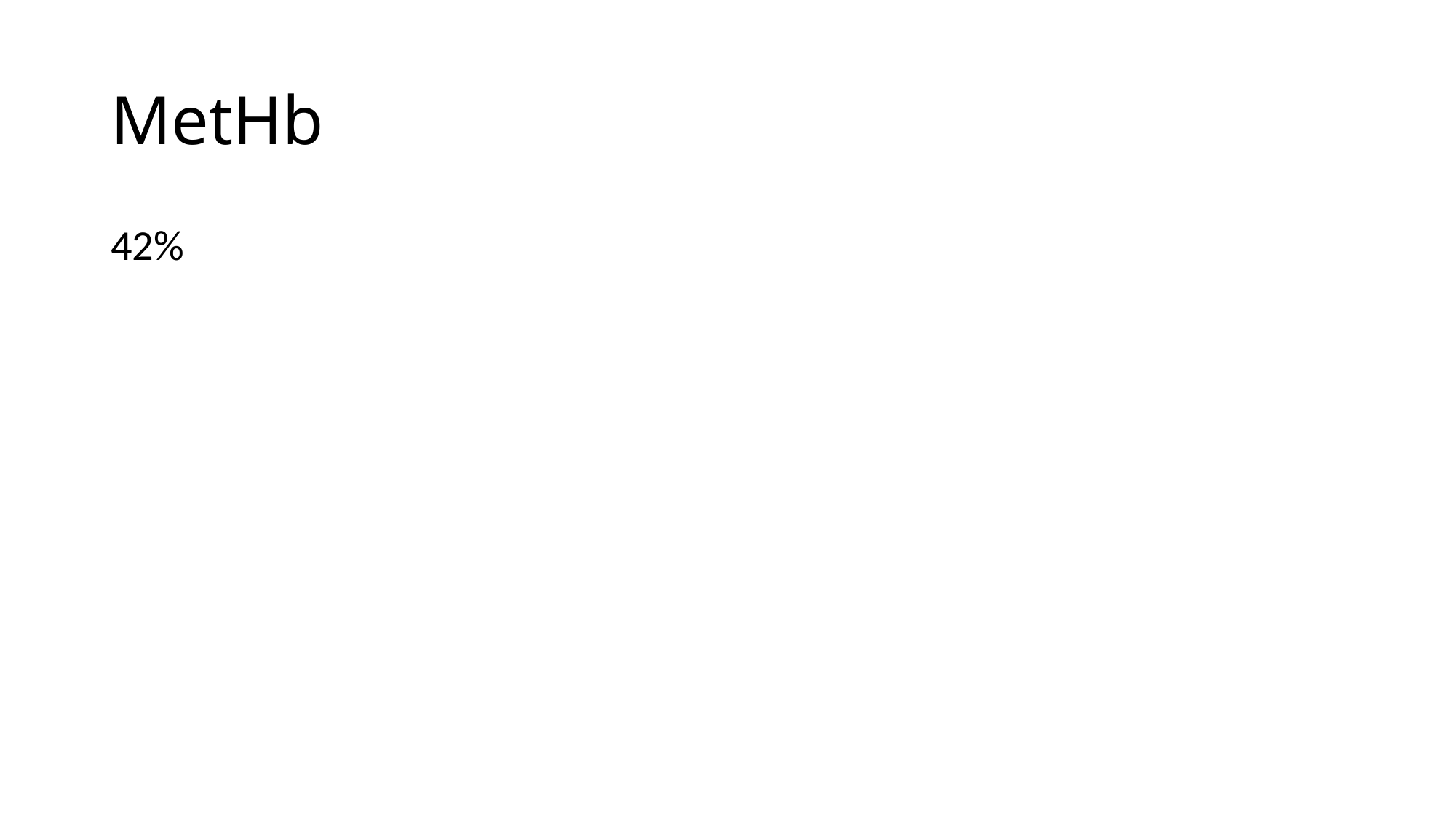

# MetHb
42%
